# Transplants of unrelated cord blood or sibling allogeneic peripheral blood stem cells/bone marrow in adolescent and young adults with chronic myeloid leukemia: comparable outcomes but better chronic GVHD-free and relapse-free survival among survivors with cord blood

**DOI:** 10.18632/oncotarget.22979

**Published:** 2017-12-05

**Authors:** Changcheng Zheng, Xiaoyu Zhu, Baolin Tang, Xuhan Zhang, Lei Zhang, Liangquan Geng, Huilan Liu, Zimin Sun

**Affiliations:** ^1^ Department of Hematology, Anhui Provincial Hospital, Anhui Medical University, Hefei, China

**Keywords:** chronic myeloid leukemia, cord blood transplantation, chronic GVHD, GVHD/relapse-free survival, adolescent and young adult

## Abstract

Adolescent and young adult (AYA) patients with hematological malignancy aged 15 to 39 years are recognized as a separate entity, and the efficacy and safety of unrelated cord blood transplantation (CBT) for chronic myeloid leukemia (CML) in AYA patients has not been reported. From March 2002 to June 2015, total of 106 CML patients received allogeneic hematopoietic cell transplantation (allo-HCT) in our center. Included in the present study were CML patients aged 15 to 39 years who received unrelated CBT or sibling allo-HCT, and 74 consecutive AYA patients with CML enrolled in this analysis. The day-100 cumulative incidences of grade 2–4 aGVHD and grade 3–4 aGVHD were similar following CBT and sibling-PBSCT/BMT. The cumulative incidences of cGVHD and extensive cGVHD were 21.7% and 5.3% in the CBT cohort, which were significantly lower than those in the sibling-PBSCT/BMT cohort (58.0% and 45.5%), respectively (*p* = 0.046, 0.008). There was no significant difference between the two cohorts in terms of transplant-related mortality (TRM), relapse, and long-term survival (overall survival and leukemia-free survival). The 5-year probability of GVHD-free/relapse-free survival (GRFS) was 47.9% and 33.4% in the CBT and the sibling-PBSCT/BMT cohorts, respectively (*p* = 0.632); among patients who survived more than 100 days after transplantation (*n* = 61), the 5-year probability of chronic GVHD-free, relapse-free survival (CRFS) was 66.2% in the CBT cohort, which was significantly higher than that in the sibling-PBSCT/BMT cohort (37.4%) (*p* = 0.037). Our study suggests that for AYA patients with CML, transplantation using unrelated CB offers comparable outcomes to sibling -PBSCT/BMT, including similar aGVHD, TRM, relapse, and long-term survival; however, from the perspective of quality of life, unrelated CBT have a lower incidence of cGVHD and a higher CRFS among survivors.

## INTRODUCTION

Tyrosine kinase inhibitors (TKIs) have remarkably improved the clinical outcomes of patients with chronic myeloid leukemia (CML), but little attention has been paid to the adolescent and young adult (AYA) group. AYA patients with hematological malignancy aged 15 to 39 years are recognized as a separate entity, with unique features of their medical and psychosocial needs which require age-appropriate treatment and care. Two retrospective clinical studies [1, 2] indicated that AYA patients with CML treated with TKIs had significantly lower complete cytogenetic and molecular response, and inferior event-free survival as compared to older patients. Although the number of allogeneic hematopoietic cell transplantation (allo-HCT) in CML has been dramatically decreased nowadays, it remains the only curative option for patients with intolerance or resistance to TKIs, or with advanced stages (accelerated phase or blast crisis, AP or BC). HLA-identical allo-HCT from a sibling donor is considered as the standard transplant model for CML, but at least two thirds of patients eligible for transplantation can not find such a donor. The development of cord blood (CB) registries and the increasing number of CB units collected allow more frequently using CB as an alternative graft source; the advantages are the immediate availability of donor cells, absence of donor risk, a lower risk of transmitting infections, and decreased graft-versus-host disease (GVHD) with preserved graft-versus-leukemia effects. Several previous reports [3–7] have demonstrated that unrelated cord blood transplantation (CBT) can be regarded as a reasonable option for CML patients requiring allo-HCT but lacking a suitable sibling donor. However, the efficacy and safety of unrelated CBT for CML in AYA patients was not reported. In this study, we retrospectively analyzed the outcomes of CML in AYA patients receiving unrelated CBT compared with those patients receiving sibling allo-HCT, with an emphasis on transplant-related complications and long-term survival, in order to explore possible survival advantages of CBT.

## RESULTS

### Engraftment

The median total nucleated cells (TNC) was 3.69 × 10^7^/kg recipient body weight in the CBT cohort and 56.8 × 10^7^/kg in the sibling-PBSCT/BMT cohort (*p* < 0.001), respectively; and the corresponding CD34+ cell dose was 2.24 × 10^5^/kg and 37.5 × 10^5^/kg recipient body weight (*p* < 0.001), respectively. PCR analysis of short tandem (STR-PCR) repeats indicated that 23 recipients (85%) from the CBT cohort and 47 recipients (100%) from the sibling-PBSCT/BMT cohort obtained primary engraftment. The median neutrophil engraftment time was 21 days (range: 14–65) in the CBT cohort and 12 days (range: 10–18) in the sibling-PBSCT/BMT cohort, and the corresponding incidence of neutrophil recovery at day 42 was 85.2% (95% CI, 63.4–94.0) and 100% in each cohort, respectively (*p* < 0.001) (Table 1). During the pre-engraftment period, four patients in the CBT cohort and 2 patients in the sibling-PBSCT/BMT cohort experienced bacterial bloodstream infections (BSIs). Despite of longer engraftment time in the CBT cohort, there was no significant difference in the incidence of BSIs between the two groups (*p* = 0.19).

**Table 1:** Patients’ and transplant characteristics

Characteristics	unrelated CBT (*n* = 27)	sibling allo-PBSCT/BMT (*n* = 47)	*p*
**Age at transplantation (years): median (range)**	27 (16–37)	30 (16–39)	0.12
**Sex : male / female**	18/ 9	32/ 15	0.95
**Disease stage at diagnosis, no (%)**			0.003
Chronic phase (CP)	10 (37.0)	36 (76.6)	
Accelerated phase (AP)	7 (26.0)	5 (10.6)	
Blast crisis (BC)	10 (37.0)	6 (12.8)	
**Pre-transplant treatment, no (%)**			0.28
Imatinib	8 (29.6)	16 (34.0)	
^†^Second-generation TKIs	6 (22.2)	5 (10.6)	
^‡^Systemic chemotherapy+TKIs	7 (25.9)	9 (19.2)	
^$^Others	6 (22.2)	17 (36.2)	
**Disease stage in transplant, no (%)**			0.19
CP (including second CP)	20 (74.1)	42 (89.4)	
AP	4 (14.8)	3 (6.4)	
BC	4 (14.8)	2 (4.2)	
**Reasons for transplant, no (%)**			
Advanced stage at diagnosis (AP/BC)	17 (63.0)	11 (23.4)	< 0.001
TKI resistance	3 (11.1)	6 (12.8)	
TKI intolerance	2 (7.4)	5 (10.6)	
£Others	5 (18.5)	25 (53.2)	
**ECOG performance status before transplant, no (%)**			0.86
0 ~ 1	19 (70.4)	35 (74.5)	
≥ 2	8 (29.6)	12 (25.5)	
**Positive recipient CMV serology prior to transplant, no (%)**	21 (77.8)	39 (83.0)	0.78
**Time from diagnosis to transplantation (months), median (range)**	13.6 (3–48)	16.2 (2–72)	0.43
**EBMT score, no (%)**			0.92
≤ 3	14 (51.9)	22 (46.8)	
< 3	8 (29.6)	14 (29.8)	
Not available	5 (18.5)	11 (23.4)	
**Donor to recipient gender, no (%)**			0.97
Female-Male	9 (33.3)	14 (29.8)	
Female-Female	4 (14.8)	6 (12.8)	
Male-Male	9 (33.3)	18 (38.3)	
Male-Female	5 (18.5)	9 (19.1)	
**HLA match (lowest), no (%)**			< 0.001
6 ⁄6	4 (14.8)	43 (91.5)	
5 ⁄6	11 (40.7)	4 (8.5)	
4 ⁄6	12 (44.4)		
**ABO compatibility, no (%)**			0.006
Match	9 (33.3)	32 (68.1)	
Major mismatch	8 (29.6)	10 (21.3)	
Minor mismatch	10 (37.0)	5 (10.6)	
**§Myeloablative Conditioning, no (%)**			< 0.001
BUCY2-based conditioning	3 (11.1)	44 (93.6)	
TBICY-based conditioning	19 (70.4)	3 (6.4)	
**Reduced intensity conditioning, no (%)**			
Fludarabine+BU+TBI+ATG/CY	5 (18.5)	0 (0)	
**GVHD prophylaxis, no (%)**			0.31
CSA+MMF	27 (100)	43 (91.5)	
CSA+MMF+MTX	0 (0)	4 (8.5)	
**Total nucleated-cell dose, median (range)** (×10^7^/kg)	3.69 (2.41–9.00)	56.8 (21.4–127.2)	< 0.001
**Total CD34+ cell dose, median (range)** (×10^5^/kg)	2.24 (0.71–7.17)	37.5 (11.1–101.0)	< 0.001
**Primary graft failure, no (%)**	4 (14.8)	0 (0)	
**Neutrophil engraftment(days), median (range)**	21 (14–65)	12 (10–18)	< 0.001
**Platelet engraftment(days), median (range)**	42 (16–121)	16 (12–50)	< 0.001
**Neutrophil engraftment at day 42 (% CumInc, 95% CI)**	85.2% (63.4–94.0)	100%	< 0.001
**Platelet engraftment at day 100 (% CumInc, 95% CI)**	79.1% (49.0–91.5)	100%	< 0.001
※**Post-transplantation TKIs use, no (%)**	5 (18.5)	8 (17.0)	0.83
**Follow-up among survivors, (months) , median (range)**	81 (18–98)	89 (19–165)	0.21

Abbreviations: CBT, cord blood transplantation; allo-PBSCT/BMT, allogeneic peripheral blood stem cells or bone marrow transplantation; TKIs, tyrosine kinase inhibitors; CMV, cytomegalovirus; BU, busulfan; CY, cyclophosphamide; TBI, total body irradiation; ATG, antithymocyte globulin; CSA, cyclosporine; MMF, mycophenolate mofetil; MTX, methotrexate; GVHD, graft-vs-host disease.

^†^Second-generation TKIs indicate nilotinib or dasatinib.

^‡^Systemic chemotherapy+TKIs indicate systemic chemotherapy combined with imatinib, nilotinib or dasatinib which is only used for patients with AP or BC stages.

^$^Others indicate interferon, hydroxyurea, or systemic chemotherapy (including low-dose arabinoside cytarabine).

^£^Others indicate patients’ willingness or physicians’ preference for transplantation.

^§^BUCY2-based conditioning includes BUCY2 plus ATG (*n* = 2), and BUCY2 plus high-dose cytarabine (*n* = 1) in the CBT cohort; and BUCY2 (*n* = 35), BUCY2 plus ATG (*n* = 4), BUCY2 plus high-dose cytarabine (*n* = 5) in the allo-PBSCT/BMT cohort. TBICY-based conditioning includes TBICY plus high-dose cytarabine (*n* = 19) in the CBT cohort, and TBICY plus fludarabine (*n* = 3) in the allo-PBSCT/BMT cohort.

^※^TKIs were used due to any level of BCR/ABL detection after transplantation.

The median platelet engraftment time was 42 days (range: 16–121) in the CBT cohort and 16 days (range: 12–50) in the sibling-PBSCT/BMT cohort, and the corresponding incidence of platelet recovery at day 100 was 79.1% (95 % CI, 49.0–91.5) and 100% in each cohort, respectively (*p* < 0.001) (Table 1).

### GVHD

**Figure 1:**
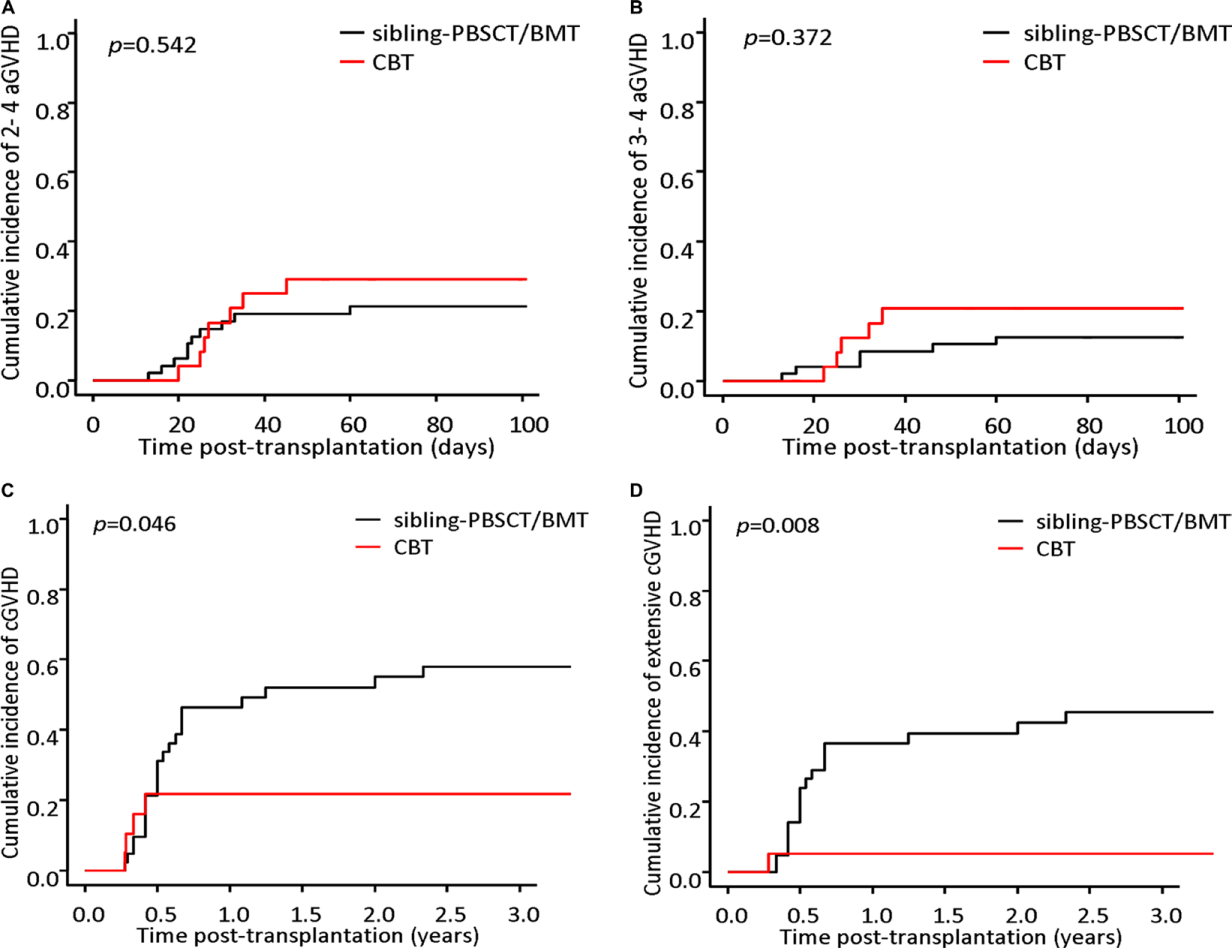
Cumulative incidence of acute GVHD and chronic GVHD The day-100 cumulative incidence of grade 2–4 aGVHD was 29.2% (95 % CI, 8.4–45.2) and 21.3% (95 % CI, 8.7–32.2) in the CBT and the sibling-PBSCT/BMT cohorts (*p* = 0.542) (**A**); and the corresponding incidence of grade 3–4 aGVHD was 20.8% (95 % CI,2.8–35.5) and 12.8% (95 % CI,2.7–21.8) for each cohort (*p* = 0.372) (**B**). The cumulative incidences of cGVHD and extensive cGVHD were 21.7% (95 % CI, 3.7–38.5) and 5.3% (95 % CI, 0–14.8) in the CBT cohort, which were significantly lower than those in the sibling-PBSCT/BMT cohort [58.0% (95 % CI, 39.0–71.1) and 45.5% (95 % CI, 27.2–59.2)] (*p* = 0.046, 0.008), respectively (**C** and **D**).

Seven patients in the CBT cohort and 10 patients in the sibling-PBSCT/BMT cohort developed grade 2–4 aGVHD, and grade 3–4 aGVHD was observed in 5 and 6 patients in each cohort, respectively. The day-100 cumulative incidence of grade 2–4 aGVHD was 29.2% (95 % CI, 8.4–45.2) and 21.3% (95 % CI, 8.7–32.2) in the CBT and the sibling-PBSCT/BMT cohorts, respectively (*p* = 0.542) ( Figure 1A); and the corresponding incidence of grade 3–4 aGVHD was 20.8% (95 % CI, 2.8–35.5) and 12.8% (95 % CI, 2.7–21.8) for each cohort (*p* = 0.372) (Figure 1B).

Total of 61 patients survived for at least 100 days after transplantation. There were 27 patients developed cGVHD (4 patients in the CBT cohort and 23 patients in the sibling-PBSCT/BMT cohort), and 19 patients developed extensive cGVHD (1 patient in the CBT cohort and 18 patients in the sibling-PBSCT/BMT cohort). The cumulative incidences of cGVHD and extensive cGVHD were 21.7% (95% CI, 3.7–38.5) and 5.3% (95% CI, 0–14.8) in the CBT cohort, which were significantly lower than those in the sibling-PBSCT/BMT cohort [58.0% (95 % CI, 39.0–71.1) and 45.5% (95 % CI, 27.2–59.2)] (*p* = 0.046, 0.008), respectively (Figure 1C and 1D).

### Transplant-related mortality and relapse

**Figure 2:**
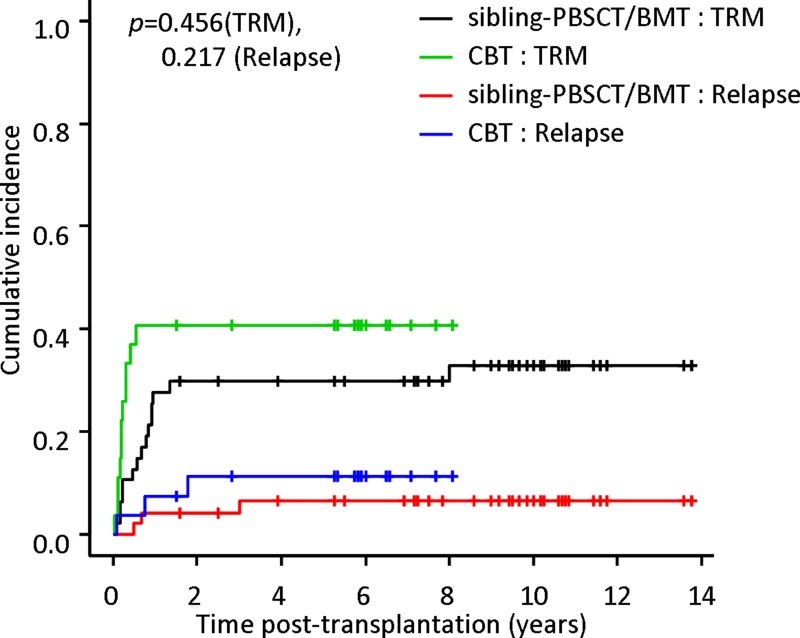
Cumulative incidences of transplant-related mortality (TRM) and relapse In the CBT cohort, the 6-month, 1-year, and 5-year cumulative incidences of TRM were 37.0% (95 % CI, 19.2–55.0), 40.7% (95 % CI, 22.1–58.6), and 40.7% (95 % CI, 22.1–58.6) compared with 12.8% (95 % CI, 5.1–24.0), 27.7% (95 % CI, 15.7–41.0), and 29.8% (95 % CI, 17.4–43.2) in the sibling-PBSCT/BMT cohort, respectively (*p* = 0.456). The 5-year cumulative incidence of relapse was 11.4% (95 % CI, 2.7–27.0) and 6.5% (95 % CI, 1.7–16.3) in the CBT and sibling-PBSCT/BMT cohorts (*p* = 0.217).

In the CBT cohort, eleven patients died due to transplant- related complications which included refractory severe aGVHD (*n* = 4), severe pneumonia (*n* =4), engraftment failure (*n* = 2), and sinusoidal obstruction syndrome (SOS) combined with intracranial hemorrhage (*n* = 1). In the sibling-PBSCT/BMT cohort, fifteen patients died of refractory extensive cGVHD (*n* = 8), severe aGVHD (*n* = 4), severe pneumonia (*n* = 3), and 1 patient died of suicide after 7 months post-transplantation. There was no significant difference in transplant-related mortality (TRM) between the two cohorts. In the CBT cohort, the 6-month, 1-year, and 5-year cumulative incidences of TRM were 37.0% (95 % CI, 19.2–55.0), 40.7% (95 % CI, 22.1–58.6), and 40.7% (95 % CI, 22.1–58.6) compared with 12.8% (95 % CI, 5.1–24.0), 27.7% (95 % CI, 15.7–41.0), and 29.8% (95 % CI, 17.4–43.2) in the sibling-PBSCT/BMT cohort, respectively (*p* = 0.456) (Figure 2).

No patients in chronic phase (CP) suffered leukemia relapse. Total of 6 patients in AP or BC experienced leukemia relapse, which included 3 patients in the CBT cohort (2 with bone marrow relapse and 1 with central nervous system relapse) and 3 patients in the sibling-PBSCT/BMT cohort (all with bone marrow relapse). The 5-year cumulative incidence of relapse was 11.4% (95 % CI, 2.7–27.0) and 6.5% (95 % CI, 1.7–16.3) in the CBT and sibling-PBSCT/BMT cohorts, respectively (*p* = 0.217) (Figure 2).

### Long-term survival

**Figure 3:**
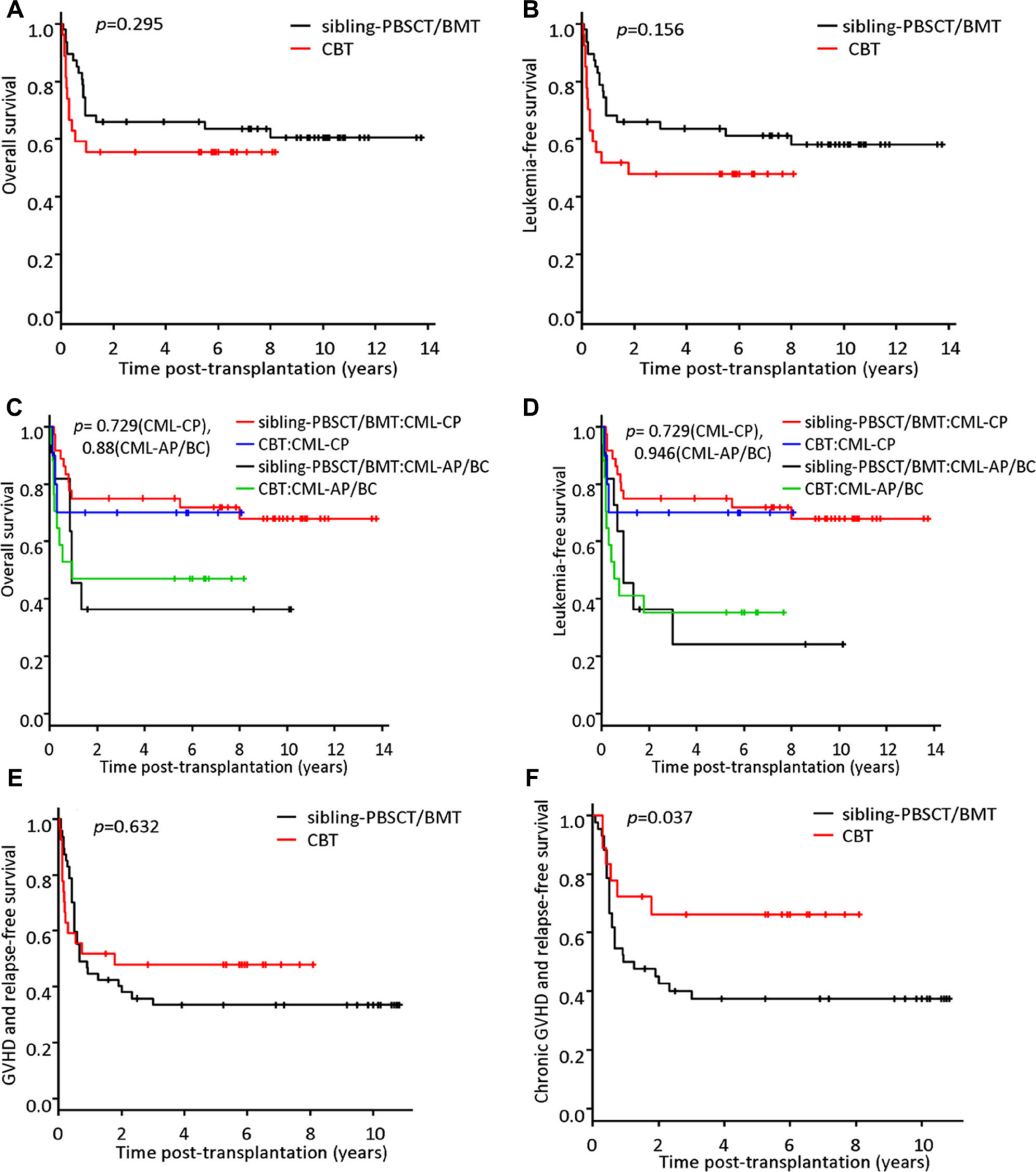
Probabilities of survival The 5-year overall survival (OS) for the CBT and the sibling-PBSCT/BMT cohorts was 55.6% (95% CI, 35.2–71.8) and 66.0% (95% CI, 50.6–77.6) (*p* = 0.295) (**A**), and the 5-year leukemia-free survival (LFS) was 47.9% (95% CI, 28.3–65.0) and 63.7% (95% CI, 48.2–75.6) (*p* = 0.156) (**B**). For subgroup analysis of patients with CML-CP, the 5-year OS was 70.0% (95% CI, 32.9–89.2) and 75.0% (95% CI, 57.5–86.1) in the CBT and the sibling-PBSCT/BMT cohorts (*p* = 0.729) (**C**), and the 5-year LFS was the same as the 5-year OS for each cohort (3d). For patients with CML-AP or BC, the 5-year OS was 47.1% (95% CI, 23.0–68.0) and 36.4% (95% CI, 14.5–62.7) in the CBT and the sibling-PBSCT/BMT cohorts, respectively (*p* = 0.88) (C), and the 5-year LFS was 35.3% (95% CI, 14.5–57.0) and 24.2% (95% CI, 13.8–52.5), respectively (*p* = 0.946) (**D**). The 5-year probability of GVHD-free/relapse-free survival (GRFS) was 47.9% (95% CI, 28.3–65.0) and 33.4% (95% CI, 20.4–47.0) in the CBT and the sibling-PBSCT/BMT cohorts, respectively (*p* = 0.632) (**E**). However, among patients who survived more than 100 days after transplantation (*n* = 61), the 5-year probability of CRFS was 66.2% (95% CI, 39.6–83.2) in the CBT cohort, which was significantly higher than that in the sibling-PBSCT/BMT cohort [37.4% (95% CI, 23.0–51.8)] (*p* = 0.037) (**F**).

The median follow-up time among survivors was 81 months (range, 18–98) and 89 months (range, 19–165) for the CBT cohort and sibling-PBSCT/BMT cohort, respectively (*p* = 0.21). The overall survival (OS) and leukemia-free survival (LFS) in the CBT cohort were similar when compared with that of the sibling-PBSCT/BMT cohort: the 5-year OS for the CBT and the sibling-PBSCT/BMT cohorts was 55.6% (95% CI, 35.2–71.8) and 66.0% (95% CI, 50.6–77.6), respectively (*p* = 0.295) (Figure 3A); and the 5-year LFS was 47.9% (95% CI, 28.3–65.0) and 63.7% (95% CI, 48.2–75.6), respectively (*p* = 0.156) (Figure 3B).

For subgroup analysis of patients with CML-CP, the 5-year OS was 70.0% (95% CI, 32.9–89.2) and 75.0% (95% CI, 57.5–86.1) in the CBT and the sibling-PBSCT/BMT cohorts, respectively (*p* = 0.729) (Figure 3C), and the 5-year LFS was the same as the 5-year OS for each cohort (Figure 3D). For patients with CML-AP or BC, the 5-year OS was 47.1% (95% CI, 23.0–68.0) and 36.4% (95% CI, 14.5–62.7) in the CBT and the sibling-PBSCT/BMT cohorts, respectively (*p* = 0.88) (Figure 3C); and the 5-year LFS was 35.3% (95% CI, 14.5–57.0) and 24.2% (95% CI, 13.8–52.5), respectively (*p* = 0.946) (Figure 3D).

Combining the events of grade 3–4 aGVHD, extensive cGVHD, relapse and death, the 5-year probability of GVHD-free/relapse-free survival (GRFS) was 47.9% (95% CI, 28.3–65.0) and 33.4% (95% CI, 20.4–47.0) in the CBT and the sibling-PBSCT/BMT cohorts, respectively(*p* = 0.632) (Figure 3E). Among patients who survived more than 100 days after transplantation (*n* = 61), the 5-year probability of chronic GVHD-free, relapse-free survival (CRFS) was 66.2% (95% CI, 39.6–83.2) in the CBT cohort, which was significantly higher than that in the sibling-PBSCT/BMT cohort [37.4% (95% CI, 23.0–51.8)] (*p* = 0.037) (Figure 3F).

## DISCUSSION

Several retrospective clinical studies [3–7] had investigated the role of unrelated CBT for the treatment of CML, and these results indicated that unrelated CBT could be used as a reasonable alternative for CML patients who needed transplantation but lacked a suitable donor. Until now, very few clinical studies have focused on the efficacy and safety of allo-HCT for CML in AYA patients. A recent CIBMTR cohort analysis [8] evaluated the outcomes of myeloablative HCT in children and young adults with CML-CP, and indicated that HLA-matched sibling donor and source of BM led to the best outcomes compared to unrelated donor and PBSC; however, this study did not cover the graft source of cord blood, and the impact of CBT on the outcomes of children and young adults CML was still a unsettled issue. In the current study, we firstly demonstrated that, for AYA patients with CML, unrelated CBT had similar incidence of aGVHD, TRM and relapse, and similar long-term survival (OS, LFS) compared to sibling-PBSCT/BMT. Moreover, it is worth mentioning that unrelated CBT had a lower incidence of cGVHD and a higher GRFS rate among patients who survived for more than 100 days after transplantation. Our data also indicated that unrelated CBT was associated with delayed neutrophil and platelet recovery compared to sibling-PBSCT/BMT. This might be due to the insufficient number of TNC and CD34+ cells in the CB graft. Nevertheless, we did not observe more bacterial or fungal infections in the CBT cohort owing to delayed neutrophil engraftment. Several strategies have been designed to accelerate neutrophil recovery, such as transplant of double CB units, injection of CB into BM, co-infusion of mesenchymal stem cells, or cytokine-mediated *ex vivo* expansion, and achieved an improvement in neutrophil or platelet engraftment.

We observed that transplantation with CB was associated with a very lower incidence of cGVHD compared to that of sibling donor (21.7% vs 58.0%, *p* = 0.046; extensive cGVHD: 5.3% vs 45.5%, *p* = 0.008). Numerous recent studies demonstrated that transplantation of CB had lower incidence and severity of cGVHD than that of related or unrelated donors. Gutman et al [9] demonstrated a significantly lower incidence of moderate to severe cGVHD following double CBT (8%) as compared with PB transplant from matched unrelated donor (44%) in hematological malignances (*p* = 0.0006). Our previous study of advanced CML (AP/ BC) [4] indicated that patients receiving CBT had slightly lower incidence of cGVHD as compared to patients receiving allo-PBSCT/BMT (19.5% vs 39.6%, *p* = 0.09). Furthermore, we also found that, for AML patients [10], CBT had a significantly reduced rate of cGVHD (13.7% vs 28.3%; *p* = 0.047) or extensive cGVHD (9.9% vs 24.1%; *p* = 0.039) compared with that of MSD. This phenomenon may be associated with 10-fold fewer T cells existed in CB, and these T cells are mostly with a naive phenotype characterized by atypical functional properties and little baseline cytotoxicity [11]. On the other hand, we speculated that more patients in the sibling cohort receiving PBSC (or PBSC plus BM) (*n* = 42, 89.4%) as a graft source may contribute to the high incidence of cGVHD, since T-cell amounts in the PBSC are higher than those in the BM or CB. Adding ATG in the conditioning to deplete T cells *in vivo* may be one of the approaches to decrease the morbidity and mortality of cGVHD following PBSCT, and a recent report from Europe [12] showed that inclusion of ATG in the conditioning resulted in a significantly lower rate of cGVHD and a higher rate of GRFS after myeloablative HLA-identical sibling PBSCT. However, other investigators found that additional ATG use might increase the disease relapse and transplant-related infections [13–16].

Among long-term relapse-free survivors after transplant, health related quality of life (HRQoL) of post-transplantation is a great concern for AYA CML patients. Most reported studies have shown that HRQoL correlates with the incidence and the severity of cGVHD, and the extensive cGVHD would have a profound negative impact on HRQoL [17–19]. GRFS is now a new composite endpoint of transplantation in current clinical trials [20], which focused on severe aGVHD, cGVHD requiring systemic treatment (extensive cGVHD), TRM, or relapse; therefore, GRFS represents a comprehensive measure of HRQoL after allo-HCT. Our previous data illustrated that [10], for AML patients, transplantation with CB had similar rates of TRM and severe aGVHD but less cGVHD and a lower risk of relapse, which translated into better GRFS as compared with sibling donor. In the present study, we found that, among patients who survived more than 100 days after transplantation, the 5-year probability of CRFS in the CBT cohort was significantly higher than that in the sibling-PBSCT/BMT cohort (66.2% vs 37.4%) (*p* = 0.037), and this indicated that AYA survivors of CML who received unrelated CBT had a better HRQoL without ongoing morbidity and experienced real recovery after transplantation.

Although German CML Study IV indicated that CML-CP patients receiving allo-HSCT had the similar survival compared with that of matched patients receiving TKIs [21], we do not recommend allo-HSCT as first-line therapy in CML-CP nowadays. Transplant should be reserved for those who are resistant or intolerant to at least one second generation TKI, or those who are in AP or BC with suitable donors. Our comparison suggests that for AYA patients with CML, transplantation using unrelated CB offers comparable outcomes to sibling -PBSCT/BMT, including similar aGVHD, TRM, relapse, and long-term survival; in addition, from the perspective of HRQoL, unrelated CBT has a lower incidence of cGVHD and a higher CRFS among survivors. However, some limitations were obvious in this study. First, this was only a retrospective study, and multivariate analyses did not performed in this study due to the small number of patients in each group. Second, the disease entity was heterogeneous, such as the fact that CML-CP and CML-AP or BC patients were mixed in this study, and more patients in the CBT cohort were associated with advanced stages (AP or BC) at first diagnosis. Third, we could not estimate the impact of post-HCT TKI use on the outcomes of transplantation, because TKI intervention was not planned for those patients before transplantation.

## MATERIALS AND METHODS

### Patient eligibility

From March 2002 to June 2015, total of 106 CML patients received allo-HCT [40 of unrelated CBT, 65 of sibling allo-HCT, and 1 of unrelated PBSCT] at Anhui Provincial Hospital (32 were previously reported [4]). Included in the present study were CML patients aged 15 to 39 years who received unrelated CBT or sibling allo-HCT. Seventy-four consecutive AYA patients with CML enrolled in this analysis, which included 27 patients receiving unrelated CBT and 47 patients receiving sibling allogeneic PBSCT or bone marrow transplantation (sibling-PBSCT/BMT) (27 received PBSC plus BM, 15 received PBSC, and 5 received BM). The baseline patient related characteristics were showed in Table 1. There were no significant differences between the CBT and the sibling-PBSCT/BMT cohorts in terms of patient age, sex, pre-transplant treatment, ECOG performance status, cytomegalovirus (CMV) serology, and EBMT score before transplant. There were more patients with advanced stages (AP or BC) at first diagnosis in the CBT cohort (*n* = 17, 63.0%) than in the sibling-PBSCT/BMT cohort (*n* = 11, 23.4%) (*p* = 0.003); however, at the time of transplantation, number of patients with advanced stages were similar between two cohorts [8 (29.6%) in the CBT cohort, and 5 (10.6%) in the sibling-PBSCT/BMT cohort] (*p* = 0.19).

### Transplant characteristics

HLA-identical sibling allo-HCT was the first selection. However, if the patient had no suitable sibling donor (HLA-identical or 1-antigen-mismatched), or there was not sufficient time to wait an unrelated donor, unrelated CBT would be performed. CB units that were serologically matched for at least 4 of 6 HLA loci and which contained a minimum count of 3 × 10^7^ ⁄ kg total nucleated cells (TNC) and/ or 1.2 × 10^5^ ⁄ kg CD34+ cells of the recipient weight before freezing. Patients without enough single cord blood unit were considered double unrelated CBT. Nineteen out of 27 patients (70.4%) received single-unit CBT, and the other 8 patients (29.6%) received double-unit CBT.

In the CBT cohort, twenty-two patients (81.5%) received a myeloablative conditioning regimen, which included TBICY plus high-dose cytarabine [total body irradiation (TBI, total 12 Gy, 4 fractions) (d-7, d-6), CY (60 mg/kg daily for 2 days) (d-3, d-2), high-dose cytarabine (2.0g/m2 every 12h for 2 days) (d-5, d-4)] *(n* = 19, 70.4%), BUCY2 [busulfan (0.8mg/kg every 6h for 4 days) (d-7~ -4) and CY (60 mg/kg daily for 2 days) (d-3, d-2)] (*n* = 3, 11.1%) [2 patients with additional antithymocyte globulin (ATG) (2.5mg/kg daily for 3 days) and 1 patient with additional high-dose cytarabine]; the remaining 5 patients (18.5%) received a reduced-intensity conditioning regimen, which consisted of fludarabine (30~ 40mg/m^2^ daily for 4 days), busulfan (0.8 mg/kg every 6 h for 2 days), low-dose TBI (3 Gy in one fraction), and cyclophosphamide (60 mg/kg daily for 1 day) (*n* = 2) or ATG (2.5 mg/kg daily for 3 days) (*n* = 3). In the sibling-PBSCT/BMT cohort, all patients received a myeloablative conditioning regimen of BUCY2 (*n* = 44, 93.6%) or TBICY plus fludarabine (*n* = 3, 6.4%); additionally, based on BUCY2, high-dose cytarabine was given to 5 patients, and ATG (2.5 mg/kg daily for 3 days) was also administered to 4 patients who received transplants from HLA-1-antigen-mismatched sibling donors. Three patients in the CBT cohort and 1 patient in the sibling-PBSCT/BMT cohort also received carmustine (250 mg/m^2^) on the first day of the conditioning in order to prevent central nervous system (CNS) recurrence.

GVHD prophylaxis was composed of cyclosporine (CSA) and mycophenolate mofetil (MMF) in all transplantations as previously described [4, 22, 23], with the exception of 4 patients in the sibling-PBSCT/BMT cohort also receiving additional short-term methotrexate (MTX).

### Definitions and statistical analyses

The definitions of neutrophil and platelet engraftment, primary graft failure, acute GVHD and chronic GVHD, transplant-related mortality (TRM), relapse, overall survival (OS), and leukemia-free survival (LFS) were previously published [22–26]. Patient-, disease-, and transplant-related variables, such as categorical variables were measured using χ^2^ test, and continuous variables were measured using Mann-Whitney *U* test between the CBT and sibling-PBSCT/BMT cohorts. The variables selected for analysis were age, gender, disease stage at diagnosis or in transplant (chronic phase or advanced stages), pre-transplant treatment, reasons for transplant, ECOG performance status, recipient CMV serology, EBMT score, donor to recipient gender, HLA match, ABO compatibility, conditioning regimens (myeloablative conditioning or reduced intensity conditioning), GVHD prophylaxis, TNC dose, and CD34+ cell dose, and post-transplantation TKIs use. The probabilities of engraftment, GVHD, TRM, and relapse were estimated by the cumulative-incidence function method with considering competing risks. The end point of OS was death of any cause, and the end point of LFS was relapse or death; the end points of GVHD-free/relapse-free survival (GRFS) were severe aGVHD (grade 3–4 aGVHD), extensive cGVHD or chronic GVHD requiring systemic treatment, relapse, or death. The probabilities of OS, LFS, and GRFS were generated by the Kaplan-Meier method. Statistical analyses were conducted using R statistical software (R Foundation for Statistical Computing, Vienna, Austria). Differences with p< 0.05 were considered significant.
